# Six Honest Serving Matters, Teaching Us All We Need to Know About Context in Knowledge Implementation? Comment on "Stakeholder Perspectives of Attributes and Features of Context Relevant to Knowledge Translation in Health Settings: A Multi-country Analysis"

**DOI:** 10.34172/ijhpm.2021.152

**Published:** 2021-11-03

**Authors:** Ann Catrine Eldh

**Affiliations:** ^1^Department of Health, Medicine and Caring Sciences, Linköping University, Linköping, Sweden.; ^2^Department of Public Health and Caring Sciences, Uppsala University, Uppsala, Sweden.

**Keywords:** Co-design, Context, Implementation, Knowledge Translation, Person-Centredness

## Abstract

While context is a vital factor in any attempt to study knowledge translation or implement evidence in healthcare, there is a need to better understand the attributes and relations that constitute context. A recent study by J. Squires et al investigates such attributes and definitions, based on 39 stakeholder interviews across Australia, Canada, the United Kingdom, and the United States. Sixteen attributes, comprising 30 elements suggested as new findings, are proposed as the basis for a framework. This commentary argues for the need to incorporate more perspectives but also suggests an initial taxonomy rather than a framework, comprising a wider range of stakeholders and an enhanced understanding of how context elements are related at different levels and how this affects implementation processes. Aligning with person-centred care, this must include not only professionals but also patients and their next of kin, as partners in shaping more evidence-based healthcare.

*“I keep six honest serving-men *

*(They taught me all I knew);*

*Their names are What and Why and When*

*And How and Where and Who.”*

 The opening of Kipling’s poem “I keep six honest serving-men”^1^ comes to mind when reading the recent publication on implementation context by Squires et al in the *International Journal of Health Policy and Management*.^2^ This effort to advance knowledge implementation, including a further understanding of context and its role in such processes and outcomes, is most welcome. Thus, it is with much interest that I embrace this study, which builds on 39 interviews across four (mainly English-speaking) countries.

 The study presents a wider range of attributes for context than previously suggested, and argues for a more robust delineation of the final 16 context characteristics. I suppose we will eventually reach a more satiated, agreed understanding of context in implementation science, and hope the findings of Squires et al can contribute to such progress. Nevertheless,the study evokes matters that are repeatedly addressed by scholars, decisionmakers, and health workers engaged in knowledge translation, for the benefit of safer and better healthcare. As implied, I address these issues with the help of the matters What, Why, When, How, Where, and Who, in order to serve further debate.

## What?

 As Squires and team note, context is not an altogether agreed-upon concept, and a number of definitions are available. Its origins indicate that context is about connections as well as how these are woven together.^3^ Consequently, one more investigation into what attributes construct the concept of context may be beneficial, particularly if triangulated with additional stakeholder perspectives, as suggested by the authors. Yet, this study represents just another investigation into *what* is connected. Like many implementation scientists, I have used contemporary tools, such as the Alberta Context Tool^4^ (56 items, arranged as 8 elements) and the Context Assessment Index (37 items, arranged in 5 factors), to capture implementation contexts.^5^ While Squires and co-authors have carefully investigated parallel and additional items, and provided further definitions, these are mainly representations of what surfaces as context, emerging as what can be named and sorted. Yet, a command of knowledge implementation as to the ‘what’ about context needs to also include what the connections are in terms of levels and relations between attributes. Thus, a primary objective to expand our understanding would be to address the layers and tiers of context. While this may be a future intention for Squires and team (or others), it is vital to provide more than just a framework in which the attributes are named and defined. Rather, progression requires a representation of what constitutes the warp and weft of context, including the significance of attributes, as well as the linkages between attributes, and levels of interdependencies.

## Why?

 The second query is why, and I address this as: why suggest one more regular nomenclature? As noted, the paper implies a framework to be the following phase, even though implementation science is by no means short of such initiatives.^6,7^ Yet another framework could of course be a steppingstone, but it is likely that the research team and the implementation community would benefit more if they could move beyond identifying and naming attributes. An alternative approach would be to deliver a joint catalogue, providing opportunities for a larger set of stakeholders to add supplementary details and help in building a taxonomy. This would be useful in both the short and long run, providing for a deeper and more thorough understanding not only of context features but also how they are woven together. Thanks to Squires and team, and earlier work by others, there is currently an opening for such an initiative. However, a subsequent taxonomy of context attributes requires (but also facilitates) the involvement of more stakeholders, as well as a more thorough investigation of ties and levels between the constituents of context. A further reflection on such an initiative is also raised below, in relation to the questions ‘Where?’ and ‘Who?’.

## When?

 Probing further into the paper, I note that there are not only the connections and layers of context to consider. Rather, there is also the issue of *when* to capture context in implementation undertakings. Squires et al^2^ indicate that a framework will guide assessments prior to or after knowledge translation initiatives. However, over the years, while working as a facilitator for knowledge implementation and as an implementation scientist, I have often wished for a better grasp of context that can envisage what enabled and/or hampered the translation of evidence-based practice guidelines into actual practice, ie, capturing context over time. This is not to say that there have not been tools available to assess context but, because of the incessant shift in context, I and others engaged in implementing evidence-based practice guidelines and policies have noticed how difficult it is to fully appreciate context and evaluate its impact. As of today, we are often limited to snapshot illustrations, by means of surveys, although interviews or observations can offer a wider narrative; interviews in particular often includes a recall of processes, yet, at times affected by wishful thinking. Triangulation of these data sources may aid the understanding of the variability of context; for instance, we found observations to uncover that particular members of staff altered compliance with the evidence-based practice guidelines in residential care which was not picked up by surveys or interviews,^8^ representing a random commitment to the implementation strategy. By and large, there is not only a need to understand what context consists of, and how the attributes are layered and related, but it is also essential to further convey the shifts in context, providing for records and reports beyond glimpses of everyday healthcare.

## How?

 How to map context is, as shown above, a delicate matter, related to both ‘what’ and ‘when’ issues. Squires et al suggest that this be addressed with their framework. I welcome any such initiative, even though I anticipate a thorough investigation of both known and so far unidentified perspectives, and the allies of attributes establishing implementation contexts. Yet, the paper generates a ‘how’ query in relation to the methods used in the study: how do we arrive at the attributes and definitions of context? Squires et al invited 39 knowledge implementation experts (researchers or practitioners) to define context, including conceptualising ‘context,’ and suggesting features of context relevant to knowledge implementation in healthcare. An inductive approach was then employed in the analysis, although the definitions were framed by three of the researchers. Also, during the final analysis phase, the analysis was reversed, and the scientists examined whether the attributes of context and their features that had emerged during the analysis were presented (per country). The authors suggest that this is a finding, although ambiguous in terms of what it represents; the reason why stakeholders did not mention a particular feature might be strictly coincidental (as noted in Limitations and Strengths). Furthermore, quotations are considered to be illustrations,^9^ but in the paper they also serve as a claim for the complexity of context and its interrelatedness – even though there is a prevailing lack of debate in relation to levels and relations. Along with the lack of further investigation, and thus understanding, of the importance of the attributes, it is possible that yet another framework on what are noted as context factors will not accomplish a further understanding. What good is a framework if it still only offers a snapshot of the context attributes? If we are to improve our understanding of a change – whether it is about to take place or has already occurred – we need further means to comprehend the processes taking place in context and how the elements and the links between them have facilitated or obstructed evidence-based practice.

## Where?

 In order to fully understand implementation contexts, we also need to recognise diversity. While the study by Squires et al includes a fair number of stakeholders, their interviews were performed in Australia, Canada, the United Kingdom, and the United States. Given that there are no notes about translations, all participants (and researchers) were presumably primarily English-speaking, representing the Anglosphere of our world. The authors suggest that this is representative enough for what is also known as the Western world, along with further studies in middle- and low-income countries. However, all in all, the world’s population is currently 7.9 billion people, and the countries where the study was conducted altogether represent a population of about 460 million. Considering that the European union alone comprises a population of close to 448 million people, with a diversity from north to south and east to west in terms of languages and cultures, healthcare structures, and values, it is important to enlarge any serious attempt to capture a full understanding of healthcare implementation contexts. This is even before we turn to middle- and low-income countries, where there is significant variation in how healthcare is organised and made accessible. Taking into account the challenges of adopting existing context measures,^10-12^ and the diversity identified across countries (eg,^13^), there is a need to encompass other perspectives. Such studies could and should comprise an investigation of both attributes and layers, enabling a more solid groundwork for definitions, within the fabric that we call context.

## Who?

 Bringing additional perspectives into a more thorough understanding, in order to develop a taxonomy, also relates to who has a say in these matters: who is a ‘stakeholder’ in knowledge translation and implementation? Contrary to the growing trend towards team orientation and partnership with clients across healthcare,^14^ including the co-design and co-production of research, Squires et al define patients as ‘receivers of healthcare services,’ which assigns clients a passive role in terms of knowledge translation. Although it is not entirely evident whether this originates from the team’s inductive analysis, ie, that this represents the experiences and/or definitions of the interviewees, or whether it is a definition composed by the researchers, it is surprising that there is no further discussion about the capacities of patients and clients, or their potential role in knowledge implementation (see eg,^15^). While many countries and healthcare organisations aim for and promote patient- or person-centred healthcare, with opportunities for people to engage in building safer and better services, the features of co-design and co-production are missing among the context attributes listed here. Even if it was not raised by the stakeholders in the study, this issue needs to be further discussed. In future attempts to address implementation context in healthcare, stakeholders should involve end-users, such as patients, clients, and their next of kin.^16^

 To conclude, context is about connections, but also about how these connections assemble and appear, when and with what effects on knowledge implementation, much like an interwoven plaid, which shifts depending on where you look, when, and how you direct your focus. To shape such a fabric, we need to collaborate. While Squires and team provide additional perspectives to our joint understanding, I anticipate that there are more nuances to be identified and defined by a larger number and variety of stakeholders.

 I look forward to our collective efforts as implementation scientists and practitioners to comprehend context in healthcare, across cultures and languages. Yet again, returning to Kipling’s poem, I find the final part of the stanza to be accommodating guidance for the future:

 “*I send them over land and sea*,

*I send them east and west*;

*But after they have worked for me*,

*I give them all a rest*.”

 In a sense, this implies that, as long as we have only listed attributes, even with suggested definitions, but with limited understanding of the ties and layers connecting them, and lacking the perspectives of a wide range of stakeholders, we had better travel both near and far, in order to address the full credentials of context. Then, but only then, can we give our serving queries on implementation context a rest.

## Ethical issues

 Not applicable.

## Competing interests

 Author declares that she has no competing interests.

## Author’s contribution

 ACE is the single author of the paper.
